# Anaesthetic Management of a Patient with Marfan Syndrome Undergoing Elective Ventral Hernia Repair

**DOI:** 10.3390/healthcare14010034

**Published:** 2025-12-23

**Authors:** Aurelijus Pūkas, Deimantė Stankutė, Jūratė Gudaitytė

**Affiliations:** Department of Anesthesiology, Lithuanian University of Health Sciences, 44307 Kaunas, Lithuania; deimante.stankute2@stud.lsmu.lt (D.S.); jurate.gudaityte@lsmu.lt (J.G.)

**Keywords:** anaesthesia, Marfan syndrome, hernioplasty

## Abstract

**Background:** Marfan syndrome is an autosomal dominant connective tissue disorder that affects multiple organ systems, with cardiovascular complications posing a major risk. With advancements in medical care and the increasing lifespan of patients with Marfan syndrome, the spectrum of medical issues has evolved. This case report highlights the complex anaesthetic management of a patient with Marfan syndrome during elective ventral hernia repair. **Case presentation:** A 37-year-old male with Marfan syndrome was admitted for elective open ventral hernia repair. Challenges included severe arterial hypertension, prior aortic valve replacement, scoliosis, and an anticipated difficult airway, as the patient presented with restricted mouth opening due to craniofacial abnormalities consistent with difficult laryngoscopy. Preoperative assessments included routine tests, echocardiography and chest X-ray. The anaesthetic management focused on specific patient positioning with head-up tilt, maintenance of haemodynamic stability with the insertion of an arterial line before the induction of anaesthesia and neuromuscular block (NMB) monitoring, followed by titrated doses of all medications. Lung ventilation strategies were specifically adjusted to address the patient’s underlying comorbidities. The patient was extubated and transferred to the recovery unit. The intraoperative and immediate postoperative periods were relatively uneventful. Dyspnea due to external pressure on the abdominal wall caused by a specific binder was treated with the release of pressure. Later postoperative recovery was complicated by hydrothorax and pneumonia, both treated successfully. **Conclusions:** This case emphasises the importance of multidisciplinary approaches and vigilant monitoring in the management of a patient with Marfan syndrome perioperatively, even for seemingly low-risk operations. Appropriate anaesthetic management helped to avoid major perioperative complications.

## 1. Introduction

Marfan Syndrome is an autosomal dominant disorder caused by a mutation in the FBN1 (fibrillin-1) gene on chromosome 15, which encodes the fibrillin protein [1]. This condition affects approximately 1 in 5000 individuals [2]. The FBN1 gene defect affects multiple organs and systems, most commonly presenting with musculoskeletal, cardiovascular, and ophthalmologic disorders [1]. The most life-threatening complications are cardiovascular, including mitral valve prolapse, aortic insufficiency, aortic root dilation, and aortic dissection [3]. With advancements in medical care and the increasing lifespan of patients with Marfan syndrome, the spectrum of medical issues has evolved. We present a clinical case to highlight the challenges and perioperative course in a patient with atypical Marfan syndrome, focusing on the specific considerations in anaesthesia management.

## 2. Case Report

The patient provided written informed consent for the preparation and publication of this case report, including the use of anonymized clinical data and imaging findings. A 37-year-old male was admitted for elective ventral hernia repair. He reported a large abdominal mass visible during exertion and coughing, which caused significant discomfort. A computed tomography (CT) scan confirmed an 8 cm ventral hernia containing small bowel. The patient had a history of Marfan syndrome and had previously undergone a life-saving Bentall procedure (composite replacement of the aortic valve and ascending aorta) performed by skilled cardiothoracic surgeons in his early childhood in the USA. He also had a history of arterial hypertension, heart failure, asthma, and glaucoma. In addition, he had spinal deformities consistent with Marfan syndrome and had undergone corrective spinal surgery.

**Figure 1:**
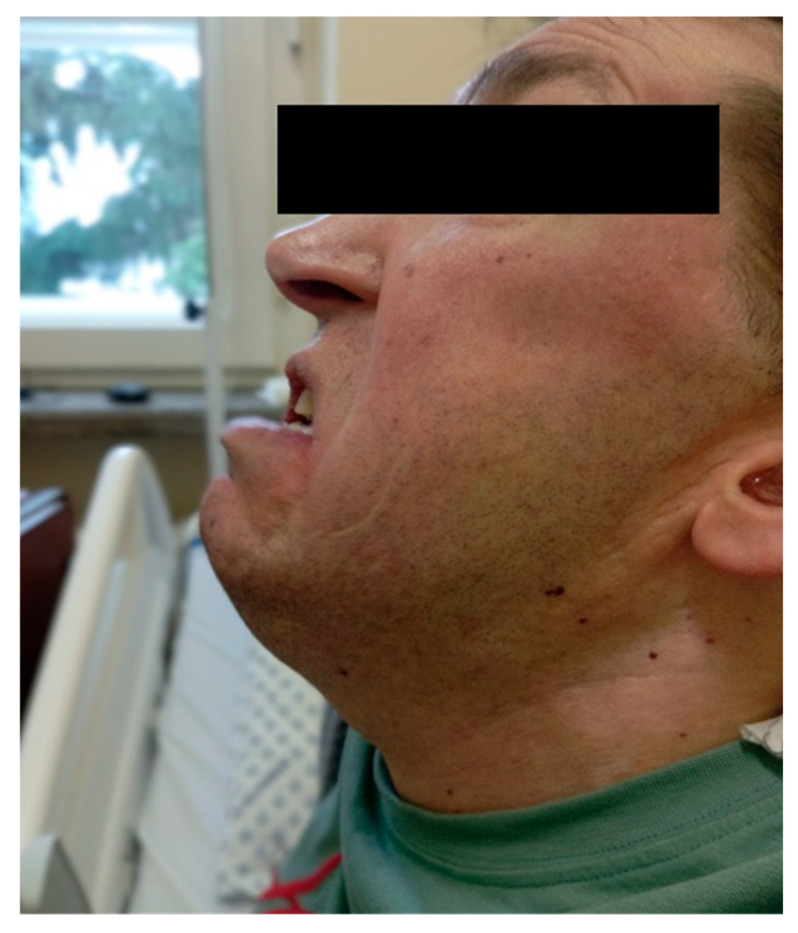
Mandibular protrusion test showing Class C limitation in the patient.

On examination, the patient had a below-average height of 160 cm, which is atypical for Marfan syndrome [4]. His weight was 63 kg, resulting in a body mass index (BMI) of 24.6 kg/m^2^, which falls within the normal range. Additional findings included reduced mandibular protrusion (Figure 1), limited mouth opening, a thyromental distance of 7 cm, a short neck, pectus carinatum, scoliosis and metatarsal joint deformities. Based on his nasopharyngeal anatomy, he was classified as Mallampati class III, and due to the restricted mouth opening, a difficult airway was anticipated.

**Figure 2:**
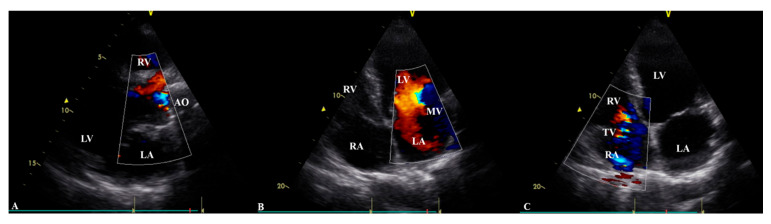
(**A**) Long-axis parasternal view showing mild (Grade I) aortic valve regurgitation. (**B**) Apical four-chamber view showing moderate-to-severe (Grade II–III) mitral valve regurgitation. (**C**) Apical four-chamber view showing moderate (Grade II) tricuspid valve. regurgitation. LV—left ventricle, LA—left atrium, RA—right atrium, RV—right ventricle, AO—aorta, MV—mitral valve, TV—tricuspid valve.

**Figure 3:**
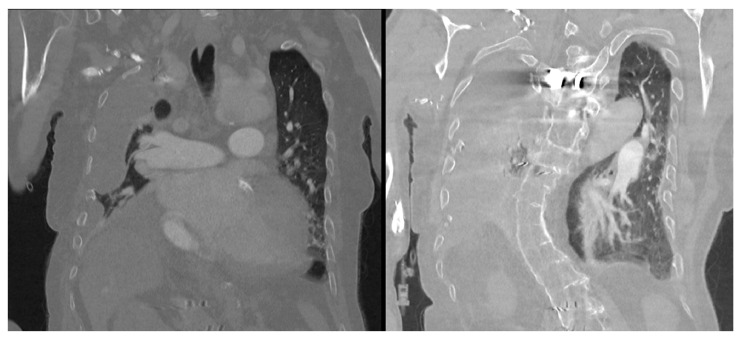
Preoperative chest CT scan showing scoliosis, metallic artefacts from prior spinal surgery, and dilatation of the right heart chambers.

Transthoracic echocardiography (Table 1) revealed a prosthetic aortic valve and an ascending aortic graft, with mild (Grade I) intraprosthetic regurgitation, eccentric left ventricular hypertrophy, and moderate-to-severe (Grade II–III) mitral valve regurgitation. The left ventricular systolic function was at the lower limit of normal, with an ejection fraction of 50%, while Grade II diastolic dysfunction was identified. The right heart chambers were dilated, with moderate (Grade II) tricuspid regurgitation observed (Table 1, Figure 2). A chest CT scan showed scoliosis, postoperative metallic artefacts from prior spinal surgery, and dilatation of the right heart chambers (Figure 3).

At the time of admission, the patient’s chronic medication regimen included warfarin 2.5–5 mg on alternating days (target INR 2.5–3.0), metoprolol 47.5 mg (half a tablet once or twice daily depending on the heart rate), zofenopril 30 mg once daily, pantoprazole 40 mg once daily, ibuprofen 400 mg as needed for musculoskeletal discomfort, and antiglaucoma eye drops.

The risks and potential complications of the current surgery were discussed in detail with the patient and his family. Anticipated perioperative risks included haemodynamic instability due to severe arterial hypertension and valvular regurgitation; the potential for acute increases in aortic wall stress given the history of an ascending aortic graft; difficult airway management due to reduced mouth opening, limited neck extension and craniofacial features; and respiratory complications related to restrictive thoracic mechanics and asthma. The risk of bleeding was addressed in the context of ongoing warfarin therapy and the presence of a mechanical aortic valve. Additional risks discussed included arrhythmias, myocardial ischaemia, pneumothorax, barotrauma during ventilation, thromboembolic events, and postoperative pulmonary complications such as atelectasis or pneumonia. These risks were explained thoroughly to the patient, and written informed consent for anaesthesia and surgery was obtained. The patient was admitted to the Department of Surgery at Kaunas Clinics of the Lithuanian University of Health Sciences Hospital, Kaunas, Lithuania, one day prior to the procedure. Complete blood count showed normal levels of haemoglobin, red blood cells (RBC), and platelets, with an elevated white blood cell (WBC) count. Biochemical analysis showed normal levels of potassium, sodium, and urea, with a slightly elevated C-reactive protein (CRP) level of 14 mg/L. Coagulation tests revealed an activated partial thromboplastin time (aPTT) within the normal range, a prolonged prothrombin time (PT), and an elevated international normalised ratio (INR) (Table 2). Given the extent of the surgical procedure, the patient’s anticoagulant therapy, and the abnormal coagulation test results, two units of packed red blood cells were cross-matched and reserved. According to the American Society of Anesthesiologists (ASA) physical status classification system, the patient was assessed as class IV. General anaesthesia with advanced monitoring, including pre-induction placement of an intra-arterial line for continuous blood pressure measurement and monitoring of neuromuscular block as well as management of a difficult airway with a videolaryngoscope and patient-specific positioning with additional pillows for a head-up tilt, was planned.

In preparation for general endotracheal anaesthesia, the patient was transferred to the operating room, and comprehensive monitoring, including non-invasive blood pressure, capnography, pulse oximetry, neuromuscular junction assessment, and electrocardiogram (ECG), was started. Due to limited neck extension observed during the preoperative evaluation, the patient’s head was positioned on an elevated head cradle. A peripheral venous catheter was inserted and preoxygenation via face mask was initiated. In accordance with the preoperative plan, left radial artery cannulation was performed under local anaesthesia following the administration of 1 mg of intravenous midazolam; the procedure was successful at the first attempt. Initial arterial pressure measurement indicated severe hypertension: 207/79 mmHg (mean arterial pressure (MAP) 115 mmHg). Neuromuscular blockade monitoring was established on the patient’s left forearm by application of a train-of-four (TOF) stimulator over the ulnar nerve and observation of the adductor pollicis muscle response. A slow administration of intravenous anaesthesia induction included fentanyl (0.1 mg, 1.6 mcg/kg), propofol (140 mg, 2.3 mg/kg), and rocuronium (30 mg, 0.5 mg/kg) titrated according to TOF monitoring results. After securing adequate mask ventilation and reaching a train-of-four (TOF) count of 0/4, endotracheal intubation was successfully performed at the second attempt using a videolaryngoscope with a D-blade and a size 7.0 endotracheal tube. The placement was confirmed by adequate capnography parameters and chest auscultation, and an orogastric tube was placed. To minimise the risk of barotrauma in this patient with a rigid and deformed chest wall, Pressure Control Ventilation Volume Guaranteed (PCV-VG) mode was selected. Ventilation settings were adjusted based on the patient’s vital signs, with a tidal volume (VT) of 400 mL, respiratory rate (RR) of 16 breaths per minute, fraction of inspired oxygen (FiO_2_) of 65%, inspiratory/expiratory time (I:E) 1:2, positive end-expiratory pressure (PEEP) of 4 cmH_2_O, and a minute ventilation of 6 L/min.

**Figure 4:**
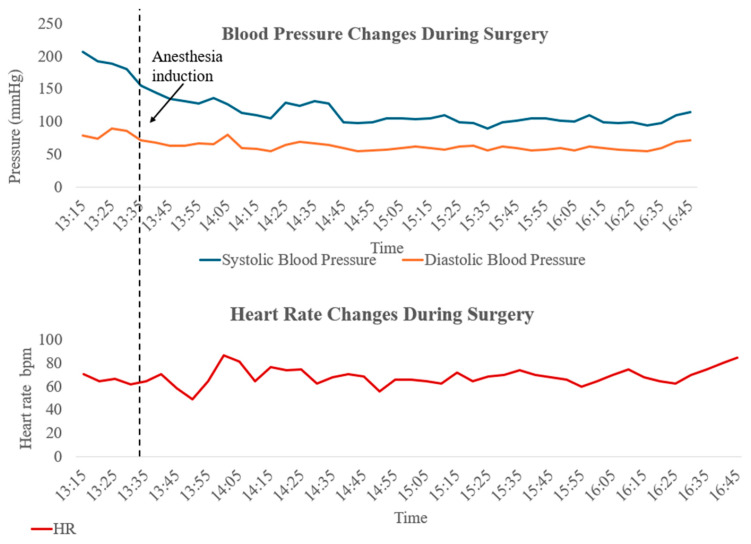
Intraoperative changes in blood pressure and heart rate.

Anaesthesia maintenance was achieved with sevoflurane (minimum alveolar concentration (MAC) 1.0), maintaining a stable sinus rhythm throughout surgery. Given the patient’s fasting state (more than six hours), a liberal fluid therapy approach was chosen, and 2000 mL of crystalloid (Ringer’s acetate), corresponding to 9.5 mL/kg/h were administered over the 3.5 h surgery. Estimated blood loss was <200 mL, so transfusion of RBCs was avoided, although 2 units of RBCs were reserved. Occasional decreases in mean arterial pressure > 20% from baseline were treated with intravenous boluses of ephedrine to a total dose of 15 mg over the course of surgery (Figure 4). Hypothermia was prevented with the use of a forced-air warming device. Mid-surgery arterial blood gas analysis indicated normal values (pH 7.4, PaCO_2_ 42.5 mmHg, PaO_2_ 215 mmHg, HCO_3_^−^ 26.2 mmol/L, lactate 0.7 mmol/L, haemoglobin 138 g/L, glucose 4.9 mmol/L, sodium 140 mmol/L, and potassium 3.6 mmol/L). Although preoperative labs revealed a potassium level of 4.6 mmol/L, 3 g of potassium chloride were given due to mild intraoperative hypokalaemia.

Intraoperative analgesia was maintained with intermittent boluses of 0.05 mg of fentanyl to a total dose of 0.2 mg. For postoperative pain relief, 1 g of paracetamol was administered at the end of surgery. Upon spontaneous return of respiration, neuromuscular blockade was fully reversed with 100 mg of sugammadex. Reversal was confirmed by TOF parameters (4/4, TOF% 93), and the patient was successfully extubated and transferred to the Post-Anaesthesia Care Unit (PACU). The intraoperative anaesthesia record is provided as Supplementary File S1. After several hours, the patient returned to the surgical ward. On the first postoperative day, recovery was complicated by dyspnea due to excessive abdominal wall compression from the external binder. Symptoms resolved after the release of abdominal wall pressure. Postoperative pain was managed with a multimodal regimen consisting of paracetamol 1 g every 6 h and ketoprophen 100 mg twice daily. Opioid analgesia (pethidine) was reserved for rescue use but was ultimately not required, as adequate pain control was achieved with non-opioid medications. Intravenous lidocaine infusion was not used, as this modality was not part of routine postoperative analgesia protocols in our centre at the time of surgery. Regional analgesic techniques, such as TAP or rectus sheath blocks, were also not performed due to concerns related to anticoagulation therapy and the potential bleeding risk associated with warfarin use as well as the patient’s anatomy—a weak abdominal wall with very thin muscular layers. Overall, pain control remained satisfactory, and no opioid-related or analgesia-related complications were observed. The in-hospital stay continued for two weeks and was complicated by a right-sided hydrothorax and pneumonia, both of which resolved with appropriate treatment. The patient was subsequently discharged from the hospital on the 13th day after surgery for continued outpatient care.

To conclude, the intraoperative course remained relatively stable, without episodes of significant cardiac arrhythmia, myocardial ischaemia or respiratory compromise, and no transfusion of blood products or coagulation agents was required. The postoperative complications of dyspnea due to abdominal binder compression, right-sided hydrothorax and pneumonia were managed successfully with conservative treatment and antibiotics. No serious cardiovascular deterioration occurred, and this supports our perioperative and anaesthetic strategy in this high-risk patient.

## 3. Discussion

This case report discusses the perioperative challenges encountered in the management of a patient with Marfan syndrome and outlines the course of perioperative care. Marfan syndrome is an autosomal dominant connective tissue disorder caused by mutations in the FBN1 gene. It affects multiple organ systems, including the cardiovascular, musculoskeletal, and ocular systems, with aortic root dilation and dissection being the most life-threatening complications. Although Marfan syndrome is classified as a rare disorder, its estimated prevalence ranges from approximately 1 in 3000 to 1 in 5000 individuals worldwide [4], which warrants that all anaesthesiologists should be familiar with the condition. Key features of the aorta in Marfan syndrome include elastic fibre degeneration, a lack of smooth muscle cells, and mucopolysaccharide deposition between the cells of the media [5]. The primary goals of anaesthetic management in patients with Marfan syndrome are the reduction in aortic wall stress and close monitoring for signs of potential aortic dissection, with prompt readiness to respond if it occurs [6].

Although opioid-free anaesthesia (OFA) and opioid-sparing anaesthesia (OSA) techniques may theoretically reduce catecholamine release and postoperative respiratory complications, a fully opioid-free approach was not selected for this patient. The decision was based on the following clinical considerations. First, the patient exhibited marked preoperative anxiety and reported poor tolerance of previous procedures performed with minimal opioid use. Second, the planned intervention involved moderate visceral manipulation, raising concern that complete opioid omission could lead to inadequate blunting of the sympathetic response. Third, higher doses of alternative agents commonly used in OFA protocols, such as dexmedetomidine or ketamine, were avoided because of the patient’s fragile cardiovascular status, including moderate-to-severe mitral and tricuspid regurgitation, a history of ascending aortic graft, and severe arterial hypertension. Therefore, a titrated low-dose opioid regimen combined with volatile anaesthesia and non-opioid analgesia was chosen to minimise endogenous catecholamine surges while maintaining haemodynamic stability [7].

Our patient exhibited numerous classical and atypical features of Marfan syndrome, including scoliosis, pectus carinatum, reduced mandibular protrusion, and notably low height, which is rare for this disease. He also had significant cardiovascular symptoms, such as prosthetic aortic valve replacement in his early childhood, tricuspid and mitral regurgitation, and severe arterial hypertension, controlled with triple antihypertensive therapy. In addition, restrictive lung physiology further complicated the case, making anaesthetic and perioperative management particularly challenging.

Although abdominal hernia repair is typically considered a low-risk surgery, in this case, it required extensive planning due to the patient’s cardiovascular status and potential airway difficulties. Due to time constraints, not all of the recommended preoperative screening tests for Marfan syndrome were completed. Cardiac magnetic resonance imaging (MRI), which would have been particularly beneficial, was not performed. However, the patient had recently undergone transthoracic echocardiography [8], which provided sufficient information [7]. In accordance with Marfan syndrome guidelines, a chest X-ray was performed one day prior to surgery to assess for pulmonary blebs, considering the increased risk of spontaneous pneumothorax.

Pulmonary function testing to evaluate restrictive lung disease, recommended for patients with significant scoliosis, could not be completed due to limited time and logistical limitations in our institution. Airway evaluation identified a high Mallampati class and reduced mouth opening, prompting preoperative planning and the use of a videolaryngoscope. To minimise the risk of temporomandibular joint dislocation, excessive traction during videolaryngoscopy was avoided, and the cardiovascular response was attenuated pharmacologically using intravenous lidocaine to reduce stress on the aortic wall.

Depth of anaesthesia monitoring using EEG-based systems—such as bispectral index (BIS), other processed EEG modalities, or density spectral array (DSA) displays—can assist in optimising hypnotic depth and reducing haemodynamic fluctuations in high-risk patients with connective tissue disorders. In our centre, such monitoring is available, but limited; however, in this particular case, it was not prioritised due to workflow and staff constraints at the time of induction, as well as the need to focus monitoring resources on the most critical concerns, namely the patient’s difficult airway, severe hypertension and significant valvular pathology. While EEG-based depth monitoring may have provided additional Supplementary Information, it is not used in our hospital on a routine basis due to financial limits. Potential benefits of monitoring the depth of anaesthesia are well appreciated, and we hope it will be included as a routine recommendation in the near future, especially for challenging patients.

There is no evidence of inherent coagulation abnormalities for Marfan syndrome and the surgery was not expected to cause major bleeding. Anticoagulation management became one of the concerns due to the patient’s mechanical aortic valve and ongoing warfarin therapy. According to the 2020 American College of Cardiology/American Heart Association (ACC/AHA) Guidelines, an INR target of 2.5 is recommended for patients with mechanical aortic valves [9]. However, in this case, a lower preoperative level of anticoagulation was considered acceptable to reduce the risk of perioperative bleeding. As a precaution, two units of cross-matched packed red blood cells were prepared in advance to treat potential anaemia, while coagulation products—fresh frozen plasma, fibrinogen concentrate and cryoprecipitate—were also immediately available if needed. As in other reported cases, haemodynamic stability was achieved through careful anaesthesia induction, intraoperative volume optimisation, and controlled extubation [10].

An arterial line was inserted to enable continuous blood pressure monitoring. Labetalol and nitroglycerin were kept readily available to manage hypertensive episodes [8]. Pressure-controlled ventilation was selected to minimise the risk of barotrauma in the context of a rigid chest wall, and neuromuscular blockade was carefully titrated based on TOF monitoring. To prevent excessive endogenous catecholamine release, small boluses of fentanyl were used for intraoperative analgesia.

When compared with previously published case reports of Marfan syndrome undergoing non-cardiac surgery, several similarities and distinctions can be noted. Other authors have described airway challenges related to craniofacial abnormalities, scoliosis and limited neck mobility, often requiring videolaryngoscopy or fibreoptic techniques, as well as cautious airway manipulation to avoid temporomandibular joint injury in patients with connective tissue laxity [11]. Similarly, invasive arterial blood pressure monitoring and careful titration of anaesthetic agents to avoid acute haemodynamic fluctuations are consistently emphasised, given the risk of aortic wall stress or dissection, particularly in patients with significant aortic root dilation or regurgitation [10]. Restrictive thoracic mechanics and scoliosis—frequently reported in Marfan syndrome—may further necessitate individualised ventilatory strategies to minimise barotrauma and optimise gas exchange [12]. These reports emphasise vast heterogeneity of Marfan manifestations and the importance of tailoring perioperative planning to the patient’s specific cardiovascular, respiratory and anatomical profile.

The generalizability of this case report is limited because of the single-patient design and heterogeneity of Marfan syndrome. Anatomical features, cardiovascular involvement, and pulmonary function can differ substantially among individuals. This requires tailored perioperative strategies. Nevertheless, several principles illustrated in this case—thorough airway assessment, anticipation of cardiovascular instability, invasive haemodynamic monitoring, cautious titration of anaesthetic agents, and lung-protective ventilation—are likely to be applicable to a broader population of patients with Marfan syndrome and similar comorbidities.

## 4. Conclusions

In conclusion, this case highlights the importance of a multidisciplinary approach in the perioperative management of patients with Marfan syndrome, particularly when they present with significant anatomical and cardiovascular abnormalities. Vigilant airway and respiratory management, appropriate patient positioning, individualised anaesthetic planning and continuous invasive haemodynamic monitoring—initiated before induction and continued into the postoperative period—are essential, even for surgeries traditionally considered low risk.

## Figures and Tables

**Table 1:** Preoperative echocardiography results.

Parameter		Value	
BSA (m^2^)	1.68	Ascending Aorta (mm)	35
LVEDD (mm)	59	Ascending Aorta Index (mm/m^2^)	20.83
LVEDD Index (mm/m^2^)	35.12	Pulmonary Flow Acceleration Time (ms)	57
LVPW Thickness (mm)	10	Aortic Valve V max (m/s)	3.1
LV Mass (g)	304.85	Aortic Valve G max (mmHg)	38.44
LV Mass Index (g/m^2^)	181.46	Mitral Valve V max (m/s)	5.4
Relative Wall Thickness	0.34	Mitral Valve G max (mmHg)	116.64
Ejection Fraction (%)	50	Tricuspid Valve V max (m/s)	3.5
IVS Thickness (mm)	14	Tricuspid Valve G max (mmHg)	49
RV Basal Diameter (mm)	60	E (cm/s)	108
LA Diameter (mm)	49	A (cm/s)	79
LA Volume (ml)	130	E/A Ratio	1.37
LA Volume Index (ml/m^2^)	130	E’ IVS (cm/s)	6.2
RA Diameter (mm)	77.38	E’ Lateral (cm/s)	12.4
S’ (cm/s)	7.3	E’ Average	9.3
		E/E’ Ratio	11.61

**Table 2:** Preoperative laboratory test results.

Parameter	Value
Haemoglobin	147 g/L
Erythrocytes	5.6 × 10^12^/L
Platelets	212 × 10^9^/L
Leukocytes	10.3 × 10^9^/L
Potassium	4.6 mmol/L
Sodium	144.6 mmol/L
Urea	6.12 mmol/L
C-reactive protein (CRP)	14 mg/L
Activated partial thromboplastin time (aPTT)	35.5 s
Prothrombin time (PT)	56%
International normalised ratio (INR)	1.48

## Data Availability

The data presented in this study are available on request from the corresponding author. (The dataset used in our case report consists exclusively of individual patient clinical information, including diagnostic imaging, laboratory results, hemodynamic charts, and perioperative monitoring data. These data contain sensitive personal health information, and despite anonymization, full public release may still risk patient re-identification due to the rarity of the condition and the detailed nature of the case.).

## Supplementary Materials

The following supporting information can be downloaded at: https://www.mdpi.com/article/10.3390/healthcare14010034/s1, File S1: Patient’s anaesthesia record. File S2: CARE checklist.

## Abbreviations

ACC	American College of Cardiology
AHA	American Heart Association
aPTT	Activated partial thromboplastin time
ASA	American Society of Anesthesiologists
BMI	Body mass index
BIS	Bispectral index
CRP	C-reactive protein
CT	Computed tomography
DSA	Density spectral array
EEG	electroencephalography
ECG	Electrocardiogram
FiO_2_	Fraction of inspired oxygen
I:E	Inspiratory to expiratory ratio
INR	International normalised ratio
MAC	Minimum alveolar concentration
MAP	Mean arterial pressure
MRI	Magnetic resonance imaging
NMB	Neuromuscular block
NSAIDs	Nonsteroidal anti-inflammatory drugs
OFA	Opioid-free anaesthesia
OSA	Opioid-sparing anaesthesia
PACU	Post-anaesthesia care unit
PCV-VG	Pressure-controlled ventilation volume guaranteed
PEEP	Positive end-expiratory pressure
PT	Prothrombin time
RBC	Red blood cell(s)
RR	Respiratory rate
TOF	Train-of-four
VT	Tidal volume
WBC	White blood cell
