# Supplementary material for: Anaesthetic Management of a Patient with Marfan Syndrome Undergoing Elective Ventral Hernia Repair
Source: Healthcare (Basel). 2025 Dec 23;14(1):34. doi: 10.3390/healthcare14010034 (PMC12785720; doi:10.3390/healthcare14010034)
Supplement: Supplementary file 1 [file healthcare-14-00034-s001.zip › Additional file S1. Patient’s anaesthesia record.pdf]

Forma Nr. **156/a**

20 24 m. 10 mėn. 03 d.

[redacted]

v., pavardė, amžius

Ligos ist. Nr. 2024-07 33839

Kraujo grupė KK 00282878 Rh

Anestezija: planinė ☒ skubi ☐ amb. ☐

SĮT

Sveikatos priežiūros įstaigos pavadinimas

ANESTEZIJOS LAPAS Nr. 1

Anestezijos būdas Endotrachėjinė anestezija

Pilvo sienos išvarža

diagnozė iki operacijos

Operacija Hernioplastica alloplastica

PREMEDIKACIJA

Užsakyti 2 vnt. EM.

PARAŠAS

DATA 2024.10.03

VAL. 13:15

Būklė iki operacijos		INKSTŲ BŪKLĖ IR SKYSČIŲ BALANSAS	KITA
**ŠIRDIES - KRAUJAGYSLIŲ SISTEMA**	**KVĖPAVIMO SISTEMA**		☐Pankreatitas
☐N.y.	☐N.y.	☐N.y.	☐Kepenų l.
☒Krūt. angina	☐Dispnėja	☐Hipovolemija	☒Tulžies p. ir takų l. [illegible]
☒Vožt. pat.	☒Astma	☐Lėtinis nepak.	☒Sąnarių l.
☐Dekomp.	☐Bronchitas	☐Oligurija	☐Vėžys
☒Hipertenzija	☐Emfizema	☐Anurija	☐Nutukimas
☐Kraujag. pat.	☐TBC	☐Poliurija	☐Kacheksija
☐MI	☐LOPS	☐Akmenligė	☐Nervų l.
☒Kita	☒Rūkymas	☒ ŠP [illegible]	☐Psichinės l.
☐Šokas	☐Kita		☒Galvos tr.
			☐Diabetas
			☐Endokrin. l.
			☒Glaukoma
			☐Antikoaguliantų terap.
			☒Kita [illegible] S. Neurosensorinis [illegible]

Ūgis/svoris 160/63	Kvėpavimo takai M III		Vaistai [illegible]
APTT 35.5	SPA 56 INR 1.48		[illegible]
Hb 144	T° N		[illegible]
Ht 44.7	AKS 166/97		NVNU
Er 5?.6	ŠSD 80		[illegible]
K 4.6	EKG SR		Alergija [illegible]
Na 144.6	KRŪT.RNTG.		
Urea 6.12	CRB-1.4		Buv. anestezijos [illegible]
Baltymas	[illegible]-10.3		
Cukrus	[illegible]-212		
Šlapimas			Valgęs prieš 28 val.
Fiz. būklė	1 2 3 (4) 5		

Laikas 13:15			14:00				15:00		16				15:45			Iš viso
N_2O l/min			1	1	1	1	1	1	1	1	1	1	10	10		
O_2 l/min			60	60	60	60	60	60	60	50	50	80	50	50		
SpO_2 %	99	99	99	98	98	98	98	98	96	96	76	96	96	97		
EtSevo				1.5	1.5	1.5	1.6	1.6	1.5	1.5	1.5	1.5	0.4	0.0		
Fentanyli				0,05				0,05								0,2 mg
Atropini		0,1														0,1 mg
Cisatracurii							5			5	5					15 mg
										3g Kef						
Infuzijos																3g
Ringer	1000									1000						2000 ml
Sugamadex													100 mg			100 mg
Kraujas																
Nukraujavimas																
Diurezė										100						100 ml

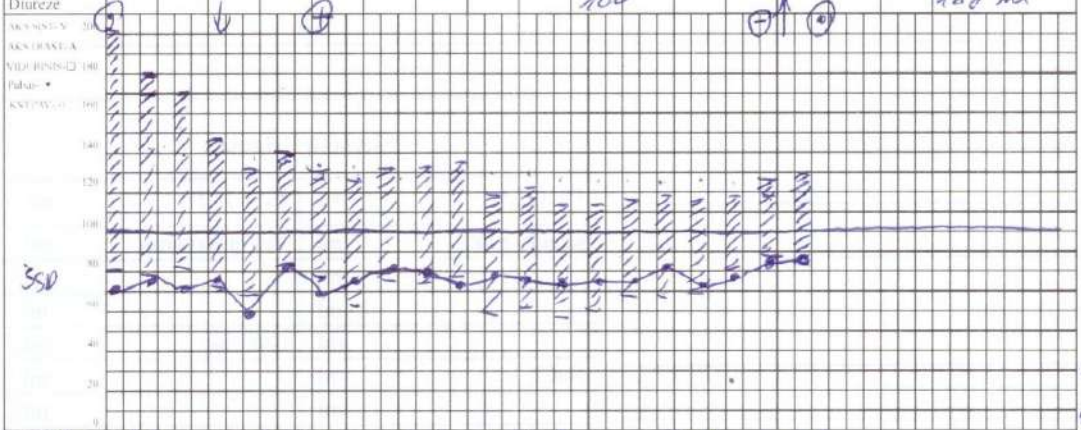

ETCO2	43 - 43 -	38 - 38	- 38 - 38	
PCV-VG				
VE	350x16 → 400x16	— 400x16 —	400x16	
ĮPŪTIMO SP	13 - 15	- 15 - 15	- 15 - 15	

Kontūras PN | Reg. anest. Spinalinė ☐ Kaudalinė ☐ | Punktuota PN Komplikacijos

Vamzdelis Nr. 7,0 | Epiduralinė ☐ Opioidai ☐ Blokas ☐ | Analgez. lygis Adatos Nr.

Rez. A. [illegible] Anesteziologas J. [illegible] Anestezistė D. [illegible] Chirurgas J. [illegible]

Monitoravimas AKS ☒ EKG ☒ SPO2 ☒ Kita ☒ [illegible]
CVS ☐ P ☒ ETCO2 ☒

Įvadinė anestezija

Midazolami 1 mg
Fentanyli 0,1 mg
Propofoli 140 mg
Rocuronium 30 mg
Cefazolini 1g 13:30

Etapai/Medikamentai

1) GFR [illegible]
2) [illegible]
3) [illegible] 100% O2 [illegible]
4) Anestezijos indukcija
5) Intubuota [illegible], ETV 7,0, [illegible], PCV.
6) [illegible] 1g i/v
Ondansetroni 8 mg i/v
7) Anestezija eigoje [illegible]
5 PAPP.
