# Supplementary material for: Anaesthetic Management of a Patient with Marfan Syndrome Undergoing Elective Ventral Hernia Repair
Source: Healthcare (Basel). 2025 Dec 23;14(1):34. doi: 10.3390/healthcare14010034 (PMC12785720; doi:10.3390/healthcare14010034)
Supplement: Supplementary file 1 [file healthcare-14-00034-s001.zip › Additional file S2. CARE checklist.pdf]

CARE case report guidelines

CARE Checklist of information to include when writing a case report

cc BY-NC-ND

✔

Topic	Item	Checklist item description	Reported on Line
**Title**	**1**	The diagnosis or intervention of primary focus followed by the words "case report"	In Title
**Key Words**	**2**	2 to 5 key words that identify diagnoses or interventions in this case report, including "case report"	In keywords section
**Abstract (no references)**	**3a**	Introduction: What is unique about this case and what does it add to the scientific literature?	In abstract – background
	**3b**	Main symptoms and/or important clinical findings	In abstract – Case presentation
	**3c**	The main diagnoses, therapeutic interventions, and outcomes.	In abstract – Case presentation
	**3d**	Conclusion—What is the main "take-away" lesson(s) from this case?	In abstract – Conclusion
**Introduction**	**4**	One or two paragraphs summarizing why this case is unique (**may include** references).	In introduction (first two paragraphs)
**Patient Information**	**5a**	De-identified patient specific information	In case report – patient description (age, sex, condition anonymised)
	**5b**	Primary concerns and symptoms of the patient	In case report – abdominal mass, discomfort
	**5c**	Medical, family, and psycho-social history including relevant genetic information	In case report – history of Marfan syndrome, cardiac surgery, hypertension, asthma, glaucoma, scoliosis
	**5d**	Relevant past interventions with outcomes	In case report – previous aortic valve replacement, spinal surgery
**Clinical Findings**	**6**	Describe significant physical examination (PE) and important clinical findings.	In case report – mandibular protrusion, Mallampati class III, scoliosis, etc.
**Timeline**	**7**	Historical and current information from this episode of care organized as a timeline	In case report_– perioperative and postoperative course
**Diagnostic Assessment**	**8a**	Diagnostic testing (such as PE, laboratory testing, imaging, surveys).	In case report CT scan, echocardiography, chest X-ray, lab tests
	**8b**	Diagnostic challenges (such as access to testing, financial, or cultural)	In case report time/logistical constraints, no pulmonary function test, no MRI
**Therapeutic Intervention**	**8c**	Diagnosis (including other diagnoses considered)	In case report ventral hernia, Marfan syndrome with comorbidities
**Follow-up and Outcomes**	**8d**	Prognosis (such as staging in oncology) where applicable	In case report/Discussion – high perioperative risk due to cardiovascular comorbidities
	**9a**	Types of therapeutic intervention (such as pharmacologic, surgical, preventive, self-care)	In case report anaesthesia, invasive monitoring, fluid therapy, analgesia
	**9b**	Administration of therapeutic intervention (such as dosage, strength, duration).	In case report- details of drug doses, ventilation settings, fluids
	**9c**	Changes in therapeutic intervention (with rationale)	In case report –

anticoagulation management adjusted, blood reserved but not transfused

10a Clinician and patient-assessed outcomes (if available .... In case report – uneventful intra-op, postoperative dyspnea, pneumonia, hydrothorax (resolved)

10b Important follow-up diagnostic and other test results.. In case report – postoperative labs, ABG, imaging

10c Intervention adherence and tolerability (How was this assessed?) .... In case report – patient tolerated interventions, extubated successfully

10d Adverse and unanticipated events.. In case report – postoperative hydrothorax, pneumonia

Discussion **11a** A scientific discussion of the strengths AND limitations associated with this case report .... In discussion – planning and monitoring strengths, limitations: incomplete pre-op screening

11b Discussion of the relevant medical literature **with references**.. In discussion – references 4–9

11c The scientific rationale for any conclusions (including assessment of possible causes).. In discussion – multidisciplinary approach, monitoring, airway strategies

11d The primary "take-away" lessons of this case report (without references) in a one paragraph conclusion................. In discussion – final paragraph

Patient Perspective **12** The patient should share their perspective in one to two paragraphs on the treatment(s) they received . . . . Not applicable

Informed Consent **13** Did the patient give informed consent? Please provide if requested . . . . **Yes** ✓ **No** ☐
